# A Monograph on Fractures: Being an Inaugural Dissertation Submitted to the Trustees and Medical Faculty of the University of Louisville, March, 1852

**Published:** 1852-07

**Authors:** James Knapp

**Affiliations:** Louisville, Ky.


Art. III.—*A Monograph on Fractures:* Being an Inaugural Dissertation submitted to the Trustees and Medical Faculty of the University of Louisville, March, 1852. By James Knapp, M. D., of Louisville, Ky.

The subject selected involves the solution of continuity of that part of the human organism, the office of which is to give form and support to the other parts of the body, to protect the more delicately organized and vital parts, as the brain and organs of the thorax, from the effects of external violence, to give attachment to the muscles, and enable man to perform the various changes of position and place, which, constituted as he is, are so essential to the continuance of his being, as well as happiness. Notwithstanding that the bones are admirably adapted to enable him to perform the various movements of will, and often to avoid danger and resist violence, yet many are subject to a great variety of accidents, some of which bring about the change which is the topic for present consideration.

We find the bones of various forms, as long, broad, and irregular, and also differing in density and power of resisting violence; some more exposed to injury from the office they perform, or the position they occupy; it is not therefore strange that, from the varied external forces that may act upon them, the muscular contractions as well as many predisposing causes, fractures should so much differ, not only in form, in the modification of treatment they demand, but in the time which nature, with the artificial aid of the surgeon, requires to effect the reparation of the injury.

Fractures have been divided into two classes—*simple* and *complicated*. A *simple* fracture indicates a solution of continuity of the bone, the bone being separated into two parts only, and not connected with an important injury of the surrounding tissues.

A *complicated* fracture is one in which the bone is broken in more places than one, is comminuted or crushed, is connected with a dislocation, or where there is an external wound, with or without protrusion of a fractured extremity, or laceration of a large blood-vessel, nerve, or injury of the brain, thoracic or abdominal viscera.

The diagnosis in most cases of fracture is not difficult; there is generally more or less distortion and inability to use the limb, if it be an extremity fractured; shortening, usually, unless the fracture be transverse, or so situated that another bone acts as a splint in preventing it; there is also pain at the seat of fracture, aggravated by motion of the injured parts, also crepitus when one fractured extremity is rubbed against the other; and there will be swelling from the extravasation of blood, or effusion of serum into the surrounding integuments. The pain and swelling, and inability to use the limb are not peculiar to fractures, therefore the distortion and crepitus are the most important and the reliable diagnostic symptoms.

The prognosis depends upon many circumstances, as the constitution, age, and habits of the patient, also upon the particular bone injured; the long bones, excepting their epiphysis, uniting much sooner than the broad and more spongy bones, the inferior maxillary, clavicle, ribs, and bones of the fore-arm uniting in the shortest period, those of the upper extremities sooner than those of the lower, the broad and spongy bones, and the epiphysis of the long bones requiring the longest time, while the olecranon, and acromion processes, the neck of the femur internal to the capsular ligament, and the patella seldom unite by bone, but by a ligamentous substance; it is said however, if the parts are kept perfectly coaptated, bony union may take place. Fractures in very old persons, and in some other bad conditions of constitution, sometimes will not unite at all; and in cases where there is too much motion allowed at the seat of fracture, bony union cannot take place, and the result will be the formation of a false joint.

The treatment of fractures consists, first, in relaxing the muscles that act in displacing the parts, and then, aided in most instances by firm yet gradual extension, and counter extension, placing their parts as nearly in their natural position as possible, after which the common roller, or in fractures of the lower extremities, the bandage of Scultetus should be applied, commencing at the extremity of the limb, sufficiently tight effectually to control the œdema and to confine the muscles, and prevent their contracting and displacing the parts. Splints, usually of wood, and as long as the fractured bone, and in some instances of greater length, having been padded with some soft material, as hair, tow or cotton, should be firmly bound to the limb, with pieces of tape or bandage, in order to prevent all motion at the seat of fracture, and retain the limb at its natural length and straightness. The dressings may require to be loosened when swelling comes on, and again to be tightened as it subsides, and if at any time an irregularity should occur, it should be immediately rectified. The parts should be kept constantly wet with cold water, or sugar of lead water, or in some instances warm water will be admissible; and quietude and the recumbent posture, in most cases, should be strictly enjoined; while the diet should be light and the medicines adapted to the symptoms of the case. In some instances, as where the patient is young and plethoric, and there is reason to believe there will be much swelling and high inflammatory action, blood should be abstracted; but in old and enfeebled constitutions, and where it is evident the vital powers will be greatly taxed previous to recovery, blood-letting should generally be avoided. In complicated fractures, the various complications will require special treatment. If there be a dislocation, it should be reduced previous to the replacing of the fractured parts; if an artery be wounded, it should be tied, or if there be an external wound, it will require appropriate dressings, or if inflammatory action should be threatened in internal organs, it will require the usual treatment. But if the thigh bone, or bones of the leg, are very much broken, as in bad forms of comminuted fracture, and more especially, if near or complicated with injury of the knee joint, or if there be extensive laceration of the soft parts, together with injury of a principal blood vessel, or nerve, it may be advisable to amputate as soon as the shock of the injury has been sufficiently recovered from, or if after any fracture of an extremity, traumatic gangrene should commence, amputation should at once be performed.

The question may now arise, how are broken bones reunited? It is evident when a fracture takes place, that not only is the osseous matter divided, but also the vessels and nerves of the bone, the medullary substance, and periosteum; and also that the surrounding textures are more or less injured; blood is effused; the reparatory process soon commences by a deposit of coagulating lymph, which stage is followed by that of fibro-cartilage, then cartilage, and finally that of ossific matter. Meanwhile, blood-vessels and nerves have become extended through the new formation, and the medullary cavity becomes re-established, the new bone having passed through precisely the same stages of formation as those of original bone.

Having thus far considered fractures in general, particular fractures will now claim attention; but in treating of the latter, my intention is to be as brief as possible, and not to dwell upon much but what is peculiar to each.

The cranium from its occupying the highest part of the osseous system, and from its being a wall of defense to a vital organ, the most wonderful of all organs, the organ of mind, serves as a very suitable point from which to commence this part of the subject.

Fracture of the *cranium* may occur where the direct force is applied, or at a distant part, may consist of a simple fissure without depression, or both tables may be depressed, or the external table in middle-aged persons may be driven into the diploë, while the internal table remains uninjured, or the internal may be fractured and depressed, and produce upon the brain all the effects of compression, while the external remains entire. I need only add, that if there be depression sufficient to produce symptoms of compression, or if it be a punctured fracture, the depressed parts should be restored, or detached spicula removed by the aid, in most cases, of the trephine.

Fracture of the *ossa nasi* will be recognized by the depression and irregularity, which in most instances, is quite manifest.

The reduction may be effected by passing a strong probe into the nostril, then with the fingers placed externally, the parts can easily be coaptated. The parts will retain their position without dressings.

Fracture of the *inferior maxillary* may occur at any portion of its extent; its most usual situation is near the middle of the horizontal ramus. The fracture will be known by the irregularity of the teeth, and laceration and bleeding of the gums. If there are loosened teeth they should be replaced; and the fractured parts brought evenly together, and if there be much tendency to redisplacement of the fracture, a tooth that is firm in its socket on one side of the fracture may be fastened to one on the other side, with a silver wire or silk ligature; the teeth of the lower jaw, should then be placed firmly against those of the upper, a compress reaching from the angle of the jaw to near the chin, should be placed beneath the jaw, a roller should then be passed by several successive turns under the jaw, up along the side of the face over the head, then several times horizontally, encircling the temple, occiput, and forehead, then by additional horizontal turns carried around the back of the neck, under the ear, over the point of the chin; a piece of roller may then be fastened to the turn at the middle of the forehead, and carried back over the top of the head, and secured to the one at the back of the neck; and pins should be inserted at each point where the roller has crossed.

Fracture of the *clavicle* is usually produced by a fall upon the elbow or shoulder. The shoulder will be depressed and fallen forward and inward, the distance between the acromion and sternum will be shortened, and there will be irregularity at the seat of fracture. The shoulder should be raised and carried outward and backward; a wedge-shaped pad should be placed in the axilla, with its large extremity upward, and confined by turns of the roller passed around the body including the pad; the elbow having been carried forward upon the chest, should be firmly supported by the roller, passed by several successive turns from the opposite shoulder over the fore-arm near the elbow, then under the elbow, around behind the chest again to the shoulder; after which the roller should be passed horizontally around the body including the arm, and the fore-arm should be placed in a sling.

Fractures of the *scapula* are of rare occurrence, yet they may take place at its neck, acromion, and coracoid processes and inferior angle. The symptoms of fracture of the neck, are depression under the acromion, and crepitus when the shoulder is moved; the part can easily be replaced, but will immediately become again displaced when the support is withdrawn. The reduction is effected by elevating the head of the humerus and pushing it outward; the position should be retained by a wedge-shaped pad in the axilla, as before directed, and the roller so applied as to keep the elbow firmly elevated, and the fore-arm should be supported.

The fracture of the *acromion* may be known by the depression at the point of the shoulder, the fractured portion being drawn downward; by the irregularity felt by tracing the spine of the scapula and by the crepitus that may be produced when the fragment is restored, and the arm rotated. The arm should be raised in order to restore the fragment, a wedge-shaped pad with its large extremity downward, should be placed between the body and the lower portion of the humerus, and the elbow should be kept well supported in order to keep the fragment sufficiently elevated, and the arm should be confined to the chest, and the fore-arm placed in a sling.

The fracture of the *coracoid* may be known by the inability to carry the arm forward and upwards, also by pressing with the finger upon the fragment. The arm should be brought forward and confined to the chest, and the fore-arm supported in the usual way.

The fracture of the *inferior angle* may be detected by fixing the point and then moving the shoulder. The arm should be carried forward upon the chest, and a compress placed against the fragment; a roller should then be carried several times around the body confining the compress against the angle, the arm should then be included by a few turns, and the fore-arm should be kept in a sling.

Fractures of the *humerus* have been divided into those of the neck, shaft, and inferior extremity.

The fracture of the neck may be recognized by the inability to raise the arm, by the irregularity that may be felt by pressing upon the fracture from the axilla, and by observing that the head rests in the glenoid cavity, and does not move with the shaft when the arm is rotated. The parts having been coaptated, and a roller applied to the entire extent of the limb, a splint should be applied, extending from the olecranon to the axilla, one from the bend of the arm to the acromion, another from the external condyle as high as the second; these should be secured to the limb by turns of the roller; a wedge-shaped pad should be placed in the axilla, with its large extremity upward if the lower fragment is drawn inward, but the reverse if it is drawn outward; the arm should be confined to the chest, and the fore arm rest in a sling.

The fracture of the shaft will require a roller, four splints, and a sling; care must be taken in this, and in fracture of the neck, that the arm is not elevated by the sling.

The fracture of the lower extremity, like other fractures near a joint, should receive much care in diagnosis. The existence of the fracture having been ascertained, the parts should be coaptated, and the limb bandaged and retained in a flexed position; two pieces of paste-board each cut at right angles, should be applied to the limb, one to the inside, the other to the outside, over which the roller should be applied, or in order to afford more firm support, angular wooden splints may be used. The angular splint with a screw will be found most convenient for varying the angle of the limb; daily passive movement of the joint should not be neglected in this or other joints near a fracture as soon as the parts have so far united as to admit of it; by this means anchylosis may frequently be prevented.

Fractures of the *fore-arm* are divided into those of the olecranon and coronoid processes of the ulna, of the shafts of the radius and ulna, and of the lower extremity of the radius.

The fracture of the olecranon may be the result of muscular contraction or external force. It may be known by the inability to straighten the arm while it can readily be flexed; by the inability of the fragment, and by the depression at the back part of the elbow, caused by the olecranon being drawn up the arm, sometimes to the distance of one or two inches. The arm should be placed in a straight positton, the fragment can then be restored; a compress should be placed against the upper portion of the fragment; a roller is to be applied from the lower extremity of the limb, and passed in the form of the figure 8 around the elbow, confining the compress against the fragment, then continued to the shoulder, in order to prevent contraction of the triceps muscle, and a straight splint suitably padded, should be confined to the fore-part of the limb.

The fracture of the *coronoid* is rarely met with, yet may occur from contraction of the brachialis muscle. There will be difficulty of bending the elbow, and the ulna will be dislocated backward. The ulna should be drawn to its place, the limb bandaged, and an angular splint applied. The angular splint should also be used in other cases of fracture of the fore-arm near the elbow. The fracture of the shafts together, or singly, may be known by the irregularity; and crepitus when the humeral extremity of the fore-arm is fixed, and the other extremity rotated. The fracture may be reduced by making pressure along the interosseous space while extension is made at the wrist, and counter-extension at the elbow; then two splints of at least three inches in width, to extend from the elbow to the points of the fingers, and padded most thickly along their middle line, in order to press the muscles into the interosseous space, should be applied, one to the palmar, the other to the dorsal surface, and confined by the roller; or the compress may be first applied, then the roller, and then the splints. The fore-arm and hand should be supported in a sling with the thumb uppermost.

The fracture of the lower extremity of the *radius* should be treated as if situated near its middle, with the addition of a compress along the dorsal, another along the palmar side, over the most projecting parts.

Fractures of the *carpal and metacarpal* bones will require a soft compress to be placed in the hollow of the hand, and a padded splint to be applied upon the palmar, another upon the dorsal side, extending from the elbow to the points of the fingers.

Fractures of the *phalanges* should be dressed with a roller, and four splints, two of which should extend from the wrist to the point of the finger.

Fractures of the *ribs* may be known by the lancinating pain when the parts are moved; by the irregularity at the seat of the fracture; and by the crepitus when the hand is placed upon the part and the patient coughs. A wide roller should be passed several times, tightly around the chest, and shoulder pieces should be attached to the turns to prevent their slipping downward.

Fracture of the *sternum* should be treated in the same way as fractured ribs.

Fracture of a *vertebra* may occur either at the body or spinous process. If the body be fractured, no attempt should be made to reduce the fracture, for such effort would probably be attended with the immediate death of the patient. Thorough antiphlogistic treatment should be early adopted; although recovery cannot be anticipated.

When the *spinous process* is fractured it should be replaced, and retained by a compress and roller; this fracture, unless complicated with injury of the spinal cord, will result favorably.

Fracture of the *os innominatum* can be produced only by great force; and is usually attended with some fatal complication. If there be displacement of the fragments the muscles acting upon them should be relaxed, and the parts replaced; a broad bandage should then be placed around the hips, and the remaining treatment adapted to the circumstances of the case.

Fracture of the *os coccygis*, or lower portion of the *sa-*

*crum*, may be reduced by passihg the finger into the rectum and pressing upon the displaced part; the bowels should be kept relaxed.

Fracture of the *femur* may take place at the neck, shaft, or inferior extremity. The fracture of the neck may be within, or external to the capsular ligament; the limb will be shortened and generally everted; and if it be drawn to its former length and rotated, crepitus will be manifest; if this extension be discontinued the limb will again become shortened and everted. A foot-board with two mortices should be provided; also two splints, each long enough to extend from the axilla to several inches below the foot; the upper extremities should be prepared like a crutch, the lower to pass through mortices of the foot board; also two long bags filled with chaff to apply to the insides of the splints, and a third to be placed between the limbs; a mattress, tapes, and a splint cloth should be arranged, and the patient placed upon them; the fracture should then be reduced, the feet confined to the foot-board, the long bags applied to the splints; and rolled in the splint cloth, the lower ends of the splints passed through the mortices and firmly secured, the upper ends bound to the shoulders; the third bag placed between the limbs, and lastly the tapes should be tied.

Fracture of the shaft has been successfully treated according to the following plan: A mattress should be provided, upon which a sheet should be placed, then pieces of tape at intervals of ten inches, over these a splint cloth a yard and a half long, and as wide as the length of the limb, over this a strong piece of paste-board to apply to the posterior part of the thigh, and to extend several inches above and below the fracture, then strips of scultetus bandage; and a large handkerchief should be placed at the upper edge of the splint cloth, another at its lower edge. Splints should also be prepared corresponding in width to the limb, one to extend from the axilla to six inches below the foot, one from the perineum to the same distance below, another to be of the length of the femur; the long splint should be formed and padded at its upper extremity like a crutch, a short distance below it should have two mortices, also one near the lower extremity, a little above a small block of wood should be attached, then two bags of suitable width and as long as the limb, should be prepared, and filled with chaff. The patient should then be placed upon the bed, and the limb placed upon the strips of bandage; one handkerchief should be used to produce extension at the ankle, the other counter extension at the groin, meanwhile the broken extremities should be placed in apposition, the bandage applied, the splints rolled in the splint cloth, the bags interposed between the splints and the limb, the extending and counter extending bands passed through the mortices and tied; then the anterior splint should be applied, the tapes tied, and a bandage passed around the pelvis, including the long splint. Care should be taken that the limb is drawn to its exact natural length, and the parts placed in their former relative position.

The fracture of the lower extremity may be treated upon the same plan as the fracture of the shaft.

Fracture of the *patella* is usually transverse, and generally caused by muscular contraction. The limb should be placed upon a padded straight splint, and kept somewhat elevated; and during the first few days means should be adopted to subdue the inflammatory action; this having been accomplished, a bandage should be placed above, another below the patella, and these should be approximated on each side of the patella by strips of tape; care being taken that the fragments are kept in close contact. The other forms of fracture of the patella may be kept approximated by the aid of the straight splint, compresses, and a roller.

Fracture of the *leg* may occur in both bones together, or in either singly. If the tibia is broken near its upper extremity the fracture should be reduced, and a scultetus bandage applied; then two splints should be prepared to extend from the upper part of the thigh to several inches below the foot, and the outside splint should be provided with a foot-piece; they should then be well padded, and confined to the sides of the limb; and the limb should be kept somewhat elevated. Fracture of both bones at their middle or lower portions, should be treated with the scultetus bandages, and two splints to extend from the knee to below the foot, and foot-piece. If only one bone is broken, but one splint need be used, and that should be placed on the side opposite the fracture. Care should be taken to keep the foot from turning too much inward or outward, and from falling below a proper line with the leg.

Fractures of the *foot* are frequently attended with severe injury of the soft parts, making amputation necessary. The fracture of the os calcis will require a bandage to be applied above the knee, and the knee to be kept flexed, and the heel elevated by a piece of tape drawn tightly from the heel of the slipper to the bandage. The other fracture of the foot will require the application of a roller, compresses, and splints so used as to preserve the proper position of the foot.

